# Factor Graph-Based Time-Synchronized Trajectory Planning for UAVs in Ground Radar Environment Simulation

**DOI:** 10.3390/s25237326

**Published:** 2025-12-02

**Authors:** Paweł Słowak, Paweł Kaczmarek, Adrian Kapski, Piotr Kaniewski

**Affiliations:** Institute of Radioelectronics, Faculty of Electronics, Military University of Technology, 00-908 Warsaw, Poland; pawel.slowak@wat.edu.pl (P.S.); pawel.kaczmarek@wat.edu.pl (P.K.); adrian.kapski@wat.edu.pl (A.K.)

**Keywords:** UAV trajectory planning, radar environment simulation, radar target emulation, factor graphs, software-defined radio (SDR)

## Abstract

The use of unmanned aerial vehicles (UAVs) as mobile sensor platforms has grown significantly in recent years, including applications where drones emulate radar targets or serve as dynamic measurement systems. This paper presents a novel approach to time-synchronized UAV trajectory planning for radar environment simulation. The proposed method considers a UAV equipped with a software-defined radio (SDR) capable of reproducing the radar signature of a simulated airborne object, e.g., a high-maneuverability or high-speed aerial platform. The UAV must follow a spatial trajectory that replicates the viewing geometry—specifically, the observation angles—of the reference target as seen from a ground-based radar. The problem is formulated within a factor graph framework, enabling joint optimization of the UAV trajectory, observation geometry, and temporal synchronization constraints. While factor graphs have been extensively used in robotics and sensor fusion, their application to trajectory planning under temporal and sensing constraints remains largely unexplored. The proposed approach enables unified optimization over space and time, ensuring that the UAV reproduces the target motion as perceived by the radar, both geometrically and with appropriate signal timing.

## 1. Introduction

Validation of modern ground-based radar systems requires reliable and repeatable measurement scenarios in which the observed target can be precisely controlled in terms of its kinematic and signal parameters. In recent years, such a mobile, reconfigurable target has increasingly been realized by unmanned aerial vehicles (UAVs) equipped with programmable software-defined radios (SDRs). These systems are capable of emulating the radar return of a wide range of airborne objects—from conventional aircraft to fast-climbing or rapidly maneuvering aerial platforms [1,2,3].

A central challenge in this approach is to ensure that the UAV trajectory is time-synchronized with the emulated target. The UAV must not only follow a feasible spatial path but also reach the correct positions at precisely the right times, so that the radar observation angles match those of the reference trajectory. Unlike range delays—which can be emulated in the SDR signal domain—the angular geometry cannot be adjusted afterwards. This means that trajectory planning must jointly account for both spatial coordinates and timestamps, extending well beyond conventional path-planning formulations [4].

To address this gap, we introduce the Time-Synchronized Trajectory Planning (TSTP) problem, formulated as a joint optimization over position and time. We adopt a factor graph representation for this task, which brings several advantages. The primary strength of factor graphs lies in their simplicity—they provide a transparent, modular way to encode different types of constraints and measurement relationships. Beyond ease of use, factor graphs offer additional benefits:

- Unified representation of heterogeneous constraints (kinematic, dynamic, geometric, and temporal) within a single optimization framework;
- Scalability, allowing new factors (e.g., additional sensors or constraints) to be seamlessly integrated;
- Robustness, thanks to well-established inference methods (e.g., Gauss–Newton solvers),
- Flexibility, enabling integration of prior information or online measurements with minimal changes to the formulation [5,6].

Within this framework, we represent the problem in terms of factor graphs that jointly encode the UAV kinematics, flight-dynamics constraints, and observation geometry, ensuring that the viewing angles remain consistent with the reference trajectory of the emulated object. The resulting formulation is solved iteratively using the Gauss–Newton method.

In this work, we analyze radar-environment simulators with emphasis on UAV-based emulation requirements, formally specify the TSTP problem in the language of factor graphs, and present both the implementation details and the architecture of the simulation module. Finally, we report experimental results and provide a comparative discussion. The results are presented for two representative motion types: a moderately maneuvering aerial platform representing smooth dynamic trajectories, and a highly dynamic high-altitude trajectory representing rapid acceleration and steep motion profiles.

To the best of our knowledge, this constitutes the first unified treatment of UAV trajectory planning synchronized in time with an emulated radar echo, combining trajectory planning, spatiotemporal synchronization, and factor graph optimization in a single framework.

The paper is structured as follows. Section 2 reviews related work, focusing on radar-environment simulators and the use of factor graphs in estimation and planning. Section 3 introduces the proposed methodology, including the formal problem formulation and the details of factor graph modeling. Section 4 presents the experimental results with the discussion, while Section 5 concludes the paper and outlines future research directions.

Beyond radar validation, the proposed factor graph-based planning mechanism represents a general-purpose framework for trajectory optimization that can be adapted to a wide range of UAV applications, including environmental monitoring, autonomous navigation, and cooperative multi-drone operations.

## 2. Related Work

### 2.1. Radar Environment Simulator

Radar Environment Simulators (RES) are key tools in radar technology, facilitating the comprehensive testing and development of radar systems under controlled conditions. Their function is indispensable, as the design, testing, and deployment of actual radar systems entail significant expenditures and are highly time-consuming. This is primarily attributable to the substantial costs associated with employing specialized platforms and the inherent risk of failing to achieve intended outcomes due to the presence or absence of specific phenomena within radar return signals or unintentional interference.

Simulators enable the replication of intricate scenarios, including signals received from unresolved targets, interference, atmospheric effects, and radar echoes with a predefined signature. This capability accelerates development cycles, reduces overall costs, and allows for a reliable evaluation of the final radar system’s performance. Furthermore, RES solutions can provide an additional source of training data for tools that use artificial intelligence (AI) and machine learning (ML) [7], as well as generate corrupted signals to validate techniques for the efficient restoration of real-world radar signals [8]. Radar simulators are also exceptionally proficient at generating simultaneous, interleaved signals for Radar Warning Receivers (RWR) [9], since the real-time identification of radar jamming signal types constitutes a critical step in radar countermeasure methods [10].

According to ref. [11], “the global radar simulator market was valued at USD 2.41 billion in 2023 and is projected to increase from USD 2.49 billion in 2024 to USD 4.21 billion by 2032”. The market is segmented into military and commercial sectors. The military segment holds the largest market share and is expected to be the fastest growing due to the emergence of modern military systems, such as network-centric and electronic warfare, which require simulators for operational testing. The commercial segment is also projected to experience significant growth, driven by the increasing global fleet of new-generation commercial aircraft and the associated demand for simulator systems for pilot training. Furthermore, key developments in autonomous cars are expected to expand this segment, including Advanced Driver Assistance Systems (ADAS), which use radar for robust environmental perception through precise range-Doppler and angular measurements [12].

A significant milestone in the development of Hardware-in-the-Loop (HIL) simulation techniques was the High Level Architecture (HLA) standard, which was developed and supported by the U.S. Defense Modeling and Simulation Office (DMSO) [13]. HIL involves connecting actual hardware to a simulation of another device or environment [14,15]. HIL includes Radio Frequency Analog-to-Digital Converter (RF ADC) hardware components in the feedback loop [16], making it particularly useful for systems that depend on accurate real-time signal processing and the generation of sensor input/output signals. For radar, this means testing the actual radar control unit in simulated environmental scenarios with negligibly low latency, on the order of tens of nanoseconds [17].

Digital Radio Frequency Memory (DRFM) systems are often used in HIL simulation for radar [18], enabling the creation of false targets and testing against electronic warfare techniques [19] under conditions similar to real-world applications. The raw radar signals generated by the RES, which serve as substitutes for radar echoes reflected from objects, can be analyzed by the developed device. The signatures of these signals—such as radar cross section (RCS), the number of scattering points, Doppler characteristics, and the object’s position in space—can be extensively modified during the measurement scenario planning stage, even for Synthetic Aperture Radar (SAR) applications [20].

In ref. [21], due to the challenges in acquiring large quantities of real-world drone data, radar return signals from the propeller blades of specific aircraft are generated by selecting appropriate parameters and incorporating the drone’s actual characteristics. These simulated signals are subsequently utilized for the training of feature-extraction and detection algorithms.

For more complex applications, the capabilities of simulators must be expanded beyond standard radar or environment modeling to include trajectory modeling that utilizes digital elevation maps (DEMs) for airborne platforms [22]. To evaluate a radar’s performance in detecting objects in low-Earth orbit (LEO), RES must, in addition to simulating real-world signals, integrate specialized techniques for compensating for the motion of LEO objects [23]. Consequently, a trajectory management and robust mission planning module is considered an essential component of modern RES.

### 2.2. Factor Graph for Estimation and Planning

Time-synchronized trajectory planning (TSTP) for UAV radar environment simulation requires an optimization framework that can jointly handle kinematic, geometric, and temporal constraints. Factor graphs (FGs) have emerged as the standard representation for large-scale nonlinear estimation, widely applied in robotics, navigation, and sensor fusion [24,25]. Their sparse structure enables efficient incremental optimization while accommodating non-Markov constraints such as temporal offsets or actuator limits [26].

Beyond estimation, trajectory planning has been reformulated as probabilistic inference on FGs, with Gaussian process priors providing smooth, continuous-time trajectories and real-time adaptability [27]. Recent advances demonstrate FG-based methods for motion planning with dynamic constraints [28] and multi-modal sensor fusion (vision, inertial, radar), improving robustness in GPS-denied environments [29,30,31]. Particularly relevant are FG back-ends for radar–inertial odometry, where Doppler and velocity factors yield reliable navigation under degraded visual conditions [32]. UAV-mounted SDR calibration methods further confirm the necessity of joint spatial–temporal optimization for accurate signal reproduction [33].

In this work, we extend these principles to formulate TSTP as a single FG optimization problem that unifies UAV trajectory estimation with synchronized SDR emissions, ensuring radar-consistent geometry and timing. Our implementation illustrates how range, azimuth, and elevation measurements can be integrated with UAV dynamics in one global optimization framework [34,35]. This structure corresponds directly to smoothing problems in which the entire trajectory is optimized jointly. Factor graphs provide a well-established mathematical framework for this class of problems, representing each measurement or kinematic prior as a factor in a global optimization graph. Their use in simultaneous localization and mapping (SLAM), multi-sensor fusion, and trajectory estimation is widely documented in recent literature [36,37,38], and our work adapts these principles to the reconstruction of a spatial–temporal trajectory that must satisfy radar-derived azimuth and elevation constraints at fixed timestamps.

### 2.3. Conventional Methods

Classical trajectory planning methods for aerial robots can be broadly divided into sampling-based, polynomial, optimal-control, filtering, and mixed-integer approaches. Sampling-based planners, such as Rapidly-Exploring Random Tree (RRT), Probabilistic Roadmap (PRM), and their optimal variants, construct feasible paths but treat geometry and timing separately [39]. Polynomial methods ensure smoothness through high-order interpolation yet rely on assumed waypoint timings [40]. Optimal control (e.g., MPC) incorporates dynamics and constraints but lacks explicit global objectives such as observation geometry [41,42]. Sequential estimators, such as the Extended Kalman Filter (EKF), are computationally efficient but cannot represent non-local constraints [43], whereas mixed-integer planners enable safety-critical decisions at high computational cost.

Conceptually our method differs from conventional UAV trajectory-planning frameworks such as polynomial-optimization methods, sampling-based (A, RRT*, RRT*-Smart) [44,45], and learning-based planners (e.g., DDPG) [46]. These methods assume a free decision space and optimize an objective function related to path length, dynamic feasibility, or smoothness. In contrast, in radar-environment simulation scenarios, the admissible trajectory is effectively unique because it is fully constrained by the underlying measurement geometry and by the timestamps of the radar-derived azimuth and elevation samples. The goal is therefore not to generate an optimal path, but to reconstruct the trajectory that fully satisfies the spatial–temporal constraints imposed by the measurement sequence.

A unifying limitation of these approaches is the decoupling of spatial and temporal dimensions. Our factor graph formulation addresses this by jointly optimizing motion and signal emission within a single framework. By incorporating position, velocity, and global delay parameters, and enforcing dynamic, geometric, and temporal constraints, the method reduces synchronization errors and enhances radar environment fidelity.

## 3. Materials and Methods

### 3.1. Problem Formulation

The goal of this work is to design a trajectory for a UAV equipped with an SDR that accurately emulates the radar return of a simulated airborne object. Unlike conventional motion planning, where the objective is primarily to reach a spatial goal while respecting kinematic and dynamic constraints, our problem requires precise synchronization of position and time. Specifically, the UAV must occupy positions in space that correspond to the radar line-of-sight geometry of a reference target trajectory, and it must do so at the specified time instants. Only by enforcing this spatiotemporal consistency can the radar echo generated onboard the UAV reproduce the angular observations of the simulated target.

Formally, let the UAV trajectory be represented as a sequence of spatiotemporally synchronized states:

(1) $$p_{k}={\left[\begin{array}{ccc} x_{k} & y_{k} & \begin{array}{cc} z_{k} & t_{k} \end{array} \end{array}\right]}^{T},$$

where the triplet ${\left[\begin{array}{ccc} x_{k} & y_{k} & z_{k} \end{array}\right]}^{T}$ denotes the spatial coordinates, and $t_{k}$ is the associated timestamp. The reference target trajectory is given as:

(2) $$r_{k}={\left[\begin{array}{ccc} x_{k}^{r} & y_{k}^{r} & \begin{array}{cc} z_{k}^{r} & t_{k}^{r} \end{array} \end{array}\right]}^{T}.$$

The UAV trajectory must satisfy two sets of conditions:

- **Kinematic feasibility:** The UAV dynamics follow either a constant-velocity (CV) or constant-acceleration (CA) model, subject to constraints on maximum velocity, acceleration, and flight-envelope limits. These constraints ensure that the planned path can be physically executed by the platform.
- **Geometric–temporal consistency:** For each step k, the UAV’s position and timestamp must align with the reference trajectory such that the observation angles from the radar site satisfy:
  (3) $$\theta \left(p_{k}\right)\approx \theta \left(r_{k}\right),$$
  (4) $$\phi \left(p_{k}\right)\approx \phi \left(r_{k}\right),$$
  where $\theta \left(\cdot \right)$ and $\phi \left(\cdot \right)$ denote the azimuth and elevation angles, respectively, as observed from the radar’s position. Importantly, both trajectory points ${\left[\begin{array}{ccc} x_{k} & y_{k} & z_{k} \end{array}\right]}^{T}$ and timestamps $t_{k}$ must be synchronized with ${\left[\begin{array}{ccc} x_{k}^{r} & y_{k}^{r} & z_{k}^{r} \end{array}\right]}^{T}$ and $t_{k}^{r}$, since spatial alignment without temporal agreement, or temporal alignment without spatial agreement, is insufficient for adequate RES functionality.

These requirements lead to the TSTP problem, formulated as a joint optimization over UAV positions and time [47]. The objective is to determine a sequence of UAV positions $p_{k}$ (and an associated SDR emissions schedule) that minimizes angular misalignment while remaining dynamically and physically feasible. The UAV must therefore reproduce the radar-centric viewing geometry of the reference object—its azimuth and elevation as perceived from the radar’s location—within prescribed angular tolerances across the entire time horizon. The motion is subject to standard kinematic constraints on velocity and acceleration. When required by sensing geometry, radar operating bounds are also enforced, including slant-range and elevation limits to avoid the cone of silence.

**Figure 1:**
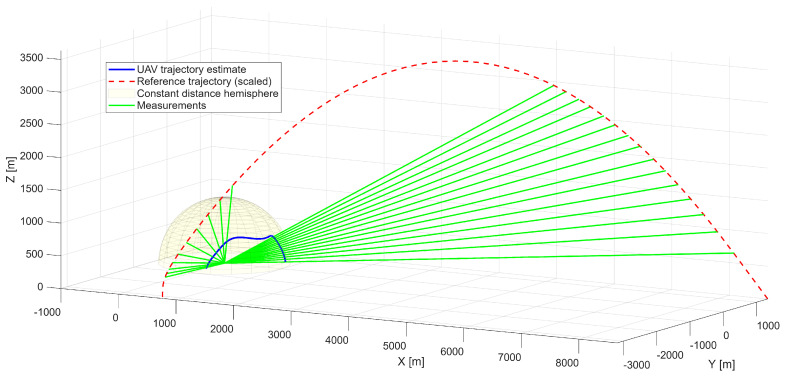
Illustration of the TSTP problem with UAV and high-speed, high-altitude motion trajectories.

The described TSTP problem is illustrated schematically in Figure 1, which highlights the UAV trajectory, the reference target trajectory, and the radar measurement geometry. For this particular implementation, it is assumed that the UAV maintains a constant distance from the radar, resulting in a fixed-radius motion envelope. This is illustrated by the highlighted hemispherical surface, which represents all feasible UAV positions.

In summary, the problem formulation differs from classical UAV planning in three ways: (i) the optimization domain includes both spatial coordinates and explicit time stamps, (ii) the objective is not merely to follow a geometric path but to reproduce the angular observations of a reference target, and (iii) the solution must guarantee feasibility with respect to UAV dynamics. This unified spatiotemporal formulation establishes the foundation for our factor graph-based implementation, which is detailed in Section 3.2.

### 3.2. Mathematical Formulation of the TSTP Problem Using Factor Graphs

A natural solution to the problem formulated in the preceding section is the factor graph approach. In this framework, each factor encodes a local relationship—such as kinematic consistency between consecutive state variables or angular alignment with the reference trajectory at a given timestamp. The global trajectory is then recovered as the maximum-likelihood estimate, obtained by solving a nonlinear least-squares optimization problem using the Gauss–Newton method [48].

#### 3.2.1. Variables

In the proposed factor graph framework, the state vector for the UAV at each discrete time step encodes the platform’s pose and motion variables. We define the state at time step k under the CV model (though it can be easily extended to CA if needed) as:

(5) $$x_{k}={\left[\begin{array}{cccccc} x_{k} & y_{k} & z_{k} & v_{x,k} & v_{y,k} & v_{z,k} \end{array}\right]}^{T},$$

where $\left(x_{k},y_{k},z_{k}\right)$ denotes position and $\left(v_{z,k},v_{y,k},v_{z,k}\right)$ the velocity components in three dimensions, representing the UAV kinematics at time step k. The discretization interval along the trajectory remains fixed and defines the implicit timestamp associated with each state. This choice eliminates the need for explicit time variables, as temporal consistency is enforced through the transition model and radar-based measurements attached to each state, as detailed in the next subsection.

#### 3.2.2. Factors

Two classes of factors are introduced: kinematic continuity factors and observation-geometry factors. Each factor contributes a local error term to the global cost function, weighted by its covariance, and collectively they enforce both motion smoothness and geometric alignment with the simulated radar target.

Kinematic continuity factors encode the assumed motion dynamics of the UAV. In navigation and localization systems, the choice of a motion model is critical and depends on the platform’s type, size, and configuration. Dynamic characteristics of different platforms may vary substantially, and this selection directly affects estimation performance. The corresponding dynamic equations are often complex, typically nonlinear, and involve dependencies on platform-specific parameters (e.g., mass, geometry, aerodynamic properties) as well as environmental conditions (e.g., air density) [49].

In the proposed method, however, the dynamics model is used solely as a generic smoothness prior within the factor graph. Therefore, simple kinematic models such as constant velocity (CV) or constant acceleration (CA) can be employed [1]. Since these models are well established and differ primarily in the order of continuity they impose, for simplicity we focus exclusively on the CV model in this work. The CV model is linear and defined as:

(6) $$x_{k}=Fx_{k-1}+w_{k-1},$$

with sampling interval ∆t. The transition matrix F is given by

(7) $$F=\left[\begin{array}{cc} I_{3\times 3} & ∆tI_{3\times 3}\\ 0_{3\times 3} & I_{3\times 3} \end{array}\right],$$

and the process noise is modeled as $w_{k}\sim N(0,Q)$, capturing deviations from the ideal CV assumption, including unmodeled accelerations, and Q is the process noise covariance matrix. This noise level controls the balance between enforcing smooth kinematic evolution and allowing the trajectory to adjust to the radar-derived constraints. It also contributes to stable numerical convergence of the Gauss–Newton optimization.

Observation-geometry factors constrain the UAV’s apparent position relative to the radar. We assume a monostatic ground radar located at the origin and a UAV subject to kinematic limits [50]. The measurement model is defined as:

(8) $$z_{k}=h\left(x_{k}\right)+v_{k}=\left[\begin{array}{c} \sqrt{x_{k}^{2}+y_{k}^{2}+z_{k}^{2}}\\ arctg\left(\frac{x_{k}}{y_{k}}\right)\\ arctg\left(\frac{z_{k}}{\sqrt{x_{k}^{2}+y_{k}^{2}}}\right) \end{array}\right]+v_{k},$$

where the measurement noise is modeled as $v_{k}∼N\left(0,R\right)$, and R is the covariance matrix of measurement errors.

We assume a fixed slant range $r_{0}$ between the radar and the UAV. In practice, this means that the UAV moves on a spherical surface centered at the radar site. This constraint simplifies the SDR’s task, as the distance between the UAV and the radar remains constant, eliminating the need for real-time computation of range-dependent signal delays in the SDR-generated radar response.

#### 3.2.3. Optimization Details

**Figure 2:**
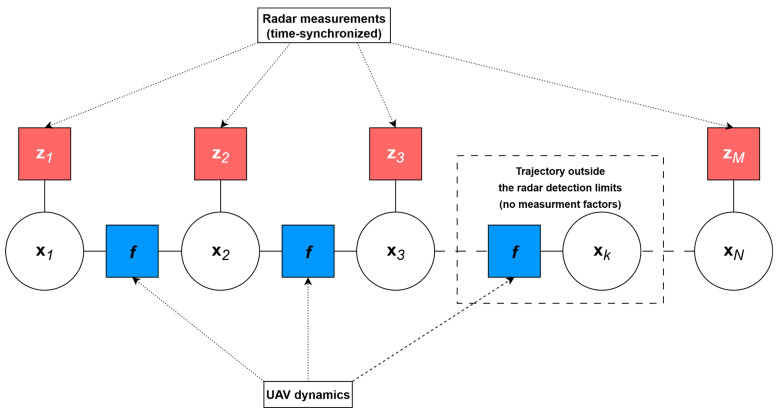
Factor graph representation of the TSTP problem for a UAV-based RES system.

The factor graph formulation described above defines a graphical structure in which each variable node corresponds to a UAV state and each factor enforces either a kinematic or an observation constraint. Figure 2 schematically illustrates this structure: variable nodes are connected by kinematic continuity factors between consecutive states, while observation-geometry factors link individual states to the corresponding radar measurements.

Proper assignment of radar measurements to the respective UAV states is critical for achieving time-synchronized trajectories. Each measurement must be associated with the correct time step k to maintain spatiotemporal alignment with the reference target.

A factor graph represents the joint probability distribution of a set of variables by expressing it as a product of multiple factors. This formulation allows the problem to be reinterpreted as an optimization task, where the objective is to minimize the sum of residuals associated with all factors. For trajectory estimation, this optimization problem can be written as:

(9) $$\hat{\chi }=\arg\underset{\chi }{\min}\sum_{k=1}^{N}\;{\left\|Fx_{k-1}-x_{k}\right\|}_{Q}^{2}+\sum_{j=1}^{M}\;{\left\|h\left(x_{k_{j}}\right)-z_{j}\right\|}_{R}^{2}.$$

where $\chi ={\left[x_{1}^{T},...,x_{N}^{T}\right]}^{T}$ denotes the stacked vector of states along the whole trajectory consisting of N waypoints, M is the number of measurements, and $x_{k_{j}}$ represents the UAV state corresponding to the j-th measurement index.

The ${\left\|\cdot \right\|}_{Q}^{2}$ and ${\left\|\cdot \right\|}_{R}^{2}$ represent squared Mahalanobis distances normalized with respect to Q and R matrices, respectively.

To simplify further calculations, we replace the terms $Fx_{k-1}-x_{k}$ and $h\left(x_{k_{j}}\right)-z_{j}$ with a unified error function $e_{i}\left(x\right)$ with its covariance matrix $\Sigma_{i}$:

(10) $$\hat{\chi }=\arg\underset{\chi }{\min}\sum_{i=1}^{M+N}\;{\left\|e_{i}\left(x_{k_{i}}\right)\right\|}_{\Sigma_{i}}^{2},$$

The optimal increment can be expressed as:

(11) $$\Delta \hat{\chi }=\arg\underset{\Delta \chi }{\min}\sum_{i=1}^{M+N}\;{\left\|e_{i}\left(x_{k_{i}}+\Delta x_{k_{i}}\right)\right\|}_{\Sigma_{i}}^{2},$$

which allows the state estimates to be refined iteratively through

(12) $${\hat{\chi }}_{j}={\hat{\chi }}_{j-1}+\Delta {\hat{\chi }}_{j}.$$

Expressing the Mahalanobis distance in terms of the Euclidean norm using the matrix square root $\Sigma^{1/2}$ and linearizing the nonlinear terms via a first-order Taylor expansion, the optimization for the incremental step can be written as:

(13) $$\Delta \hat{\chi }=\arg\underset{\Delta \chi }{\min}\sum_{i=1}^{M+N}\;{\left\|{\Sigma_{i}}^{-T/2}e_{i}(x_{k_{i}})+{\Sigma_{i}}^{-T/2}J_{i}\Delta x_{k_{i}}\right\|}^{2},$$

where $J_{i}$ is the Jacobian of the i-th error function. For the dynamic constraint the error term $Fx_{k-1}-x_{k}$ depends linearly on the state variables, and $J_{i}=\left[\begin{array}{cc} F & -I_{6\times 6} \end{array}\right]$. For the measurement factor, the error term $h\left(x_{k_{j}}\right)-z_{j}$ involves a non-linear function $h\left(\cdot \right)$, and the Jacobian $J_{i}=H\left(x_{k_{i}}\right)$ at the k-th waypoint is given by

(14) $$H\left(x_{k_{i}}\right)=\frac{\partial h\left(x_{k_{i}}\right)}{\partial x_{k_{i}}}=\left[\begin{array}{cc} H_{p}\left(x_{k_{i}}\right) & 0_{3\times 3} \end{array}\right],$$

where

(15) $$H_{p}\left(x_{k_{i}}\right)=\left[\begin{array}{ccc} \frac{x_{k_{i}}}{D_{k_{i}}} & \frac{y_{k_{i}}}{D_{k_{i}}} & \frac{z_{k_{i}}}{D_{k_{i}}}\\ \frac{y_{k_{i}}}{d_{k_{i}}} & -\frac{x_{k_{i}}}{d_{k_{i}}} & 0\\ -\frac{x_{k_{i}}z_{k_{i}}}{d_{k_{i}}D_{k_{i}}^{2}} & -\frac{y_{k_{i}}z_{k_{i}}}{d_{k_{i}}D_{k_{i}}^{2}} & \frac{d_{k_{i}}}{D_{k_{i}}^{2}} \end{array}\right],$$

where $D_{k_{i}}$ and $d_{k_{i}}$ compactly represent the distance to the waypoint and its projection onto the xy-plane, respectively:

(16) $$D_{k_{i}}=\sqrt{x_{k_{i}}^{2}+y_{k_{i}}^{2}+z_{k_{i}}^{2}},\;\;\;\;\;\;\;\;d_{k_{i}}=\sqrt{x_{k_{i}}^{2}+y_{k_{i}}^{2}}.$$

By defining two new structures: a sparse matrix A:

(17) $$A=\left[\begin{array}{ccc} {\Sigma_{1}}^{-T/2}J_{1} & \ldots & \ldots \\ \vdots & {\Sigma_{i}}^{-T/2}J_{i} & \vdots \\ \ldots & \ldots & {\Sigma_{M+N}}^{-T/2}J_{M+N} \end{array}\right],$$

and a stacked vector b:

(18) $$b=\left[\begin{array}{c} {\Sigma_{1}}^{-T/2}e_{1}(x_{k_{1}})\\ \begin{array}{c} \vdots \\ {\Sigma_{i}}^{-T/2}e_{i}(x_{k_{i}})\\ \vdots \end{array}\\ {\Sigma_{M+N}}^{-T/2}e_{M+N}(x_{k_{M+N}}) \end{array}\right],$$

and using A and b, the optimization criterion can be formulated as a typical least-squares problem:

(19) $$\Delta \hat{\chi }=\arg\underset{\Delta \chi }{\min}{\left\|A\Delta \chi +b\right\|}^{2}.$$

The sparsity of J stems from the locality of factors: each motion factor connects only consecutive states, while each measurement factor connects only a single state. This structure enables efficient solution using sparse QR decomposition which is described in detail in [51].

## 4. Results

### 4.1. Simulation Setup

The evaluation of the proposed factor graph-based trajectory planning method was carried out within a MATLAB 2025b simulation framework designed to replicate the observation conditions of a ground-based radar. The environment enables the generation of reference trajectories for aerial vehicles and the simulation of corresponding radar measurements [52].

The simulation workflow consisted of five main stages: (i) generation of a reference trajectory, (ii) initialization of a naive straight-line trajectory serving as a baseline, (iii) construction of the factor graph incorporating both dynamic and measurement constraints, (iv) nonlinear least-squares optimization using Gauss–Newton iterations until convergence, and (v) evaluation of the recovered trajectory, including analysis of the UAV’s kinematic parameters.

The simulation was configured using a sampling interval of 0.05 s, with 6000 trajectory points generated for each run. The radar sensor was positioned at the origin (0,0,0). The process noise covariance matrix Q corresponds to the standard white noise model for CV state transition [49]:

(20) $$Q=\left[\begin{array}{cccccc} \frac{∆t^{3}}{3} & 0 & 0 & \frac{∆t^{2}}{2} & 0 & 0\\ 0 & \frac{∆t^{3}}{3} & 0 & 0 & \frac{∆t^{2}}{2} & 0\\ 0 & 0 & \frac{∆t^{3}}{3} & 0 & 0 & \frac{∆t^{2}}{2}\\ \frac{∆t^{2}}{2} & 0 & 0 & ∆t & 0 & 0\\ 0 & \frac{∆t^{2}}{2} & 0 & 0 & ∆t & 0\\ 0 & 0 & \frac{∆t^{2}}{2} & 0 & 0 & ∆t \end{array}\right]q,$$

where q denotes the spectral noise density (units: $m^{2}s^{-3}$) used to scale the process noise intensity. The value of q was selected through tuning to ensure stable optimization and appropriate flexibility of the kinematic prior. In the simulations it was set to $q=2\;m^{2}\;s^{-3}$.

This formulation provides a standard way of introducing process noise into a CV motion model and ensures numerically stable behavior of the Gauss–Newton iterations.

The measurement model incorporates a diagonal measurement noise covariance matrix:

(21) $$R=\left[\begin{array}{ccc} \sigma_{r}^{2} & 0 & 0\\ 0 & \sigma_{az}^{2} & 0\\ 0 & 0 & \sigma_{el}^{2} \end{array}\right],$$

where $\sigma_{r}$, $\sigma_{az}$, and $\sigma_{el}$ represent the standard deviations of the range, azimuth and elevation measurements, respectively. In the simulations, these values were set to $\sigma_{r}=0.1\;m$, $\sigma_{az}=0.1\;deg$, and $\sigma_{el}=0.2\;deg$. This diagonal structure reflects the assumption of mutually uncorrelated measurement errors in range, azimuth, and elevation. The factor graph optimization employs a Gauss–Newton solver with a maximum of six iterations, which was empirically found sufficient to ensure convergence while maintaining computational efficiency.

The developed simulation tool was validated by analyzing its performance for two representative types of aerial objects. The corresponding results, illustrating the accuracy and robustness of the proposed approach under different motion profiles, are presented and discussed in the subsequent part of this section.

### 4.2. Moderate-Maneuvering Aerial Trajectory Simulation

**Figure 3:**
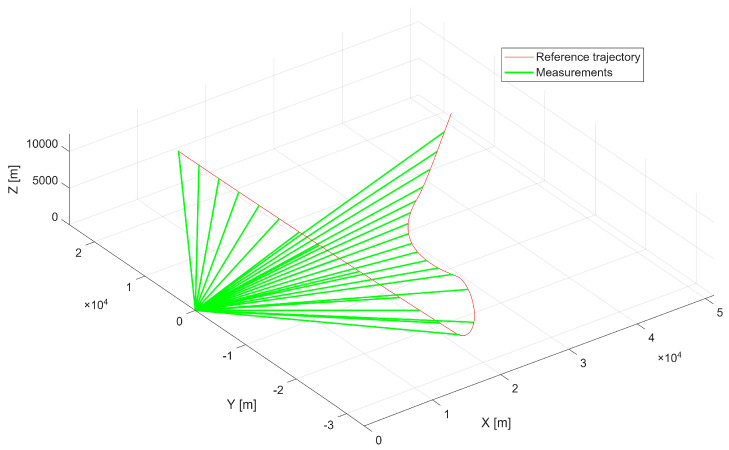
First scenario reference trajectory with radar measurements.

The first scenario represents a maneuvering aerial platform with a maximum linear velocity of 480 m/s, executing turns that generate centripetal accelerations of up to approximately 5g. Figure 3 presents the reference trajectory together with radar measurements, which serve as inputs to the optimization process.

**Figure 4:**
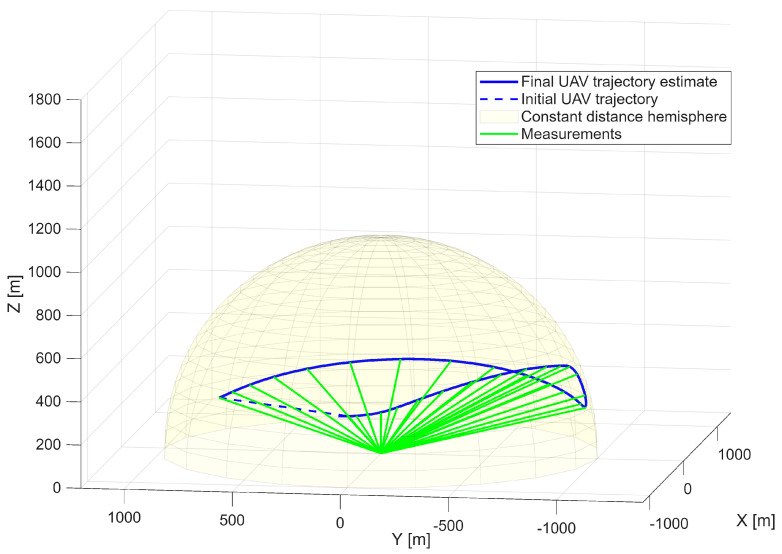
Optimized UAV trajectory on the surface of the constant distance hemisphere—first scenario.

The analyzed maneuver involves a climbing trajectory combined with a half-roll, representative of high-agility platforms. This configuration provides a test case reflecting the dynamics of a rapid course-changing maneuver. The optimized UAV trajectory is presented in Figure 4, demonstrating successful reproduction of the reference geometry. The UAV maintains a constant distance from the radar while accurately replicating the angular observation geometry of the target.

**Figure 5:**
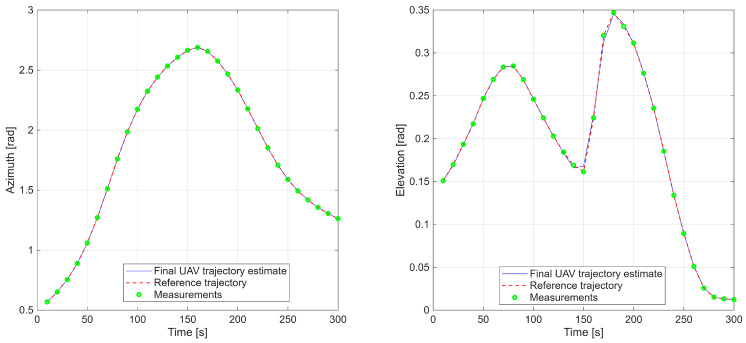
First scenario with azimuth and elevation time series comparing reference trajectory, radar data, and UAV estimates.

A complementary plot (Figure 5) compares the reference trajectory and radar-derived measurements with the optimized UAV path in the radar coordinate system, highlighting close spatial and temporal correspondence.

**Figure 6:**
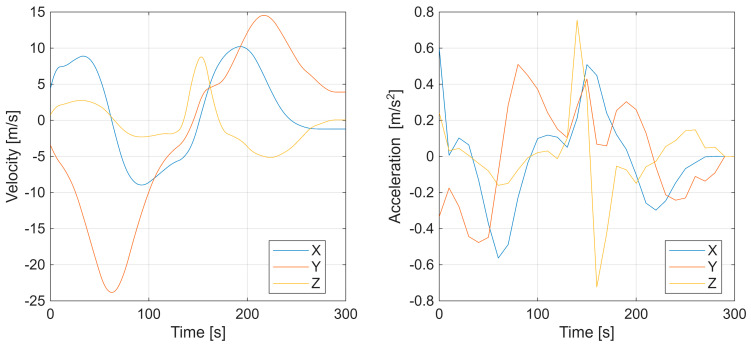
First scenario with UAV velocity and acceleration components along the X, Y, and Z axes.

Additional results, in Figure 6, depict the UAV velocity and acceleration components along the X, Y, and Z axes, confirming smooth dynamic behavior and continuity of motion. Overall, the first experiment demonstrates that the factor graph-based optimization effectively reconstructs both spatial and temporal characteristics of a maneuvering target.

Table 1 summarizes the angular reconstruction accuracy obtained in scenario 1, reported in terms of the root-mean-square error (RMSE) for both azimuth and elevation angles.

### 4.3. High-Speed, Steep-Climb Trajectory Simulation

**Figure 7:**
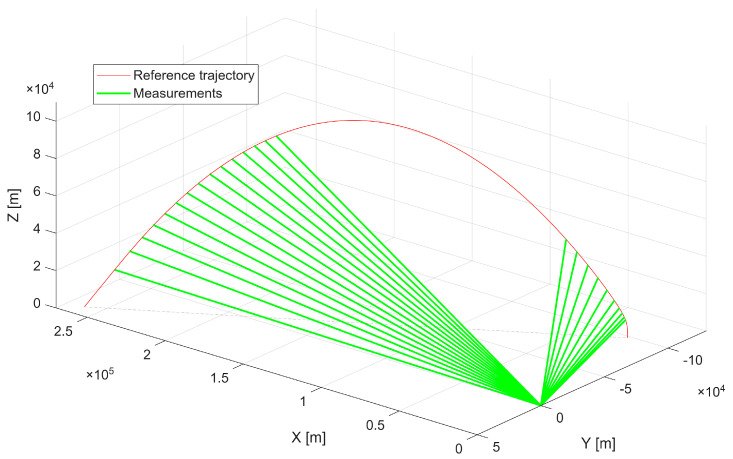
Second scenario reference trajectory with radar measurements.

The second scenario represents a high-speed, high-altitude trajectory exhibiting rapid acceleration and steep vertical motion. The figures for this experiment are structured analogously to those of the first case, allowing direct comparison between scenarios. Figure 7 presents the reference trajectory alongside radar measurements. It is noteworthy that certain segments of the trajectory exceed the radar’s coverage in elevation angle, introducing additional challenges for trajectory reconstruction and synchronization.

**Figure 8:**
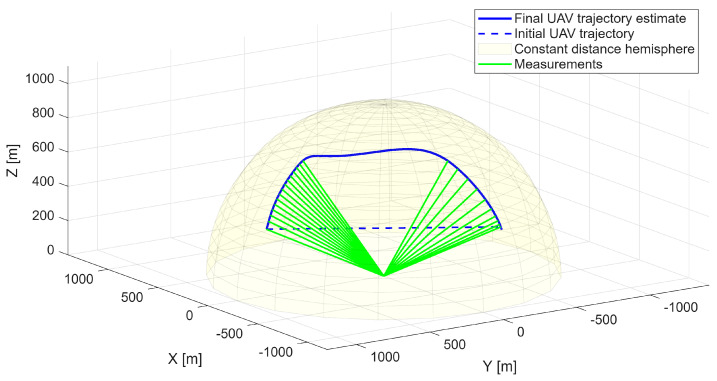
Optimized UAV trajectory on the surface of the constant distance hemisphere—second scenario.

Figure 8 illustrates the optimized UAV trajectory. Outside the radar’s elevation coverage, the UAV is not required to fully replicate the reference trajectory; nevertheless, it maintains accurate spatial alignment with the radar observations.

**Figure 9:**
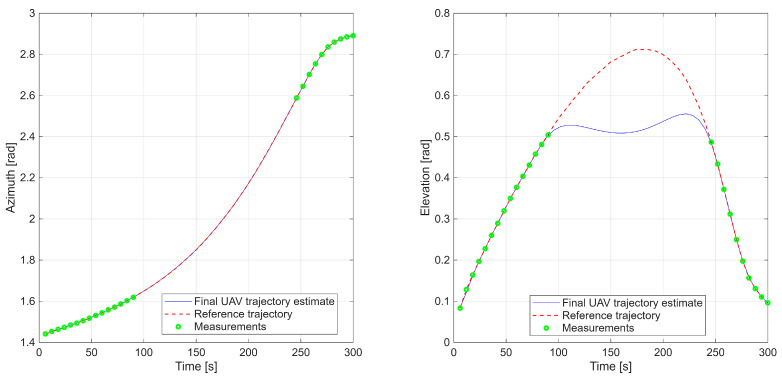
Second scenario with azimuth and elevation time series comparing reference trajectory, radar data, and UAV estimates.

Next, Figure 9 presents a plot comparing the reference and estimated angles of radar observation, illustrating spatial alignment and temporal synchronization of the UAV trajectory and the reference target. Additionally, the elevation–versus–time plot indicates that the UAV does not unnecessarily increase its altitude when the target is located inside the radar’s cone of silence.

**Figure 10:**
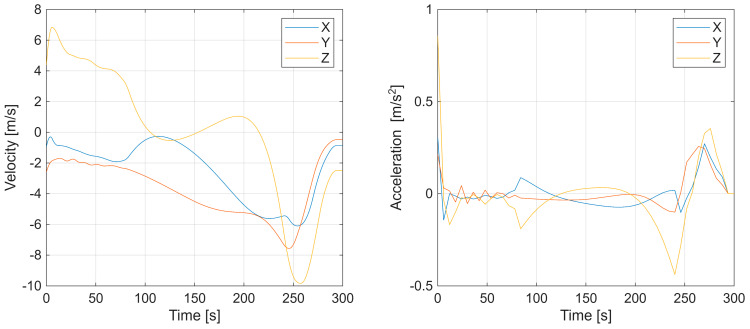
Second scenario with UAV velocity and acceleration components along the X, Y, and Z axes.

The velocity and acceleration profiles in the X, Y, and Z directions (Figure 10) indicate stable dynamic performance and maintain trajectory continuity.

Table 2 presents the angular reconstruction accuracy achieved in scenario 2, expressed as the root-mean-square error (RMSE) for both azimuth and elevation angles.

Collectively, these results confirm the capability of the factor graph-based framework to generate UAV trajectories within the RES system, enabling accurate reproduction of radar observations in both spatial and temporal domains. The reconstructed trajectories demonstrate high accuracy, robustness, and consistency across various target types. These findings substantiate the effectiveness of the proposed approach as a key component of the RES radar evaluation methodology.

In scenario 2, the simulated target moves at a higher velocity and follows a trajectory with a more pronounced vertical component. As a result, the elevation profile exhibits faster temporal variation than in scenario 1, while the larger range still reduces the rate of change of the azimuth angle. These differing angular dynamics explain the mixed reconstruction behavior observed across the two scenarios: scenario 2 yields more accurate azimuth reconstruction, whereas scenario 1 provides slightly better elevation accuracy. In both cases, however, the factor graph optimization converges reliably within six Gauss–Newton iterations, and the resulting angular errors remain within the tolerances defined by the radar’s intrinsic measurement accuracy.

## 5. Conclusions

In this work, a factor graph-based framework for time-synchronized UAV trajectory planning in radar environment simulation has been presented and evaluated. The results demonstrate that the proposed approach reliably reproduces the spatial and temporal characteristics of reference targets, including both moderate-maneuvering and high-dynamic aerial trajectories. The trajectories generated by the optimization framework maintain continuity in position, velocity, and acceleration while ensuring precise angular alignment with the radar’s observation geometry.

The proposed method is not a general trajectory-planning algorithm, but a measurement-driven reconstruction framework grounded in nonlinear smoothing and factor graph estimation. In radar-environment simulation, the desired UAV trajectory is fully specified by a sequence of time-indexed azimuth and elevation measurements that describe the apparent motion of a simulated target. As a consequence, the problem is not one of optimizing a free trajectory, but of determining a state sequence that simultaneously satisfies measurement-derived geometric constraints and temporal synchronization.

The framework proved to be robust and effective, producing trajectories that are consistent with reference measurements and well-suited for emulating complex radar returns. Its modular and scalable design makes it particularly well-suited for integration into operational systems. Indeed, this approach is planned for implementation within the radar evaluation system currently under development at the Military University of Technology, providing a versatile tool for testing and validating modern radar sensors and trajectory-planning algorithms for various civilian and research applications.

Future work will focus on several directions to further enhance system capabilities. These include extending the framework to multi-UAV coordination for simultaneous emulation of multiple targets. Additionally, further research will explore the use of factor graphs for online UAV control within a model predictive control (MPC) framework.

Although the proposed method is intended for offline generation of trajectories used in radar-environment simulation (RES), where the trajectory must reproduce a predefined sequence of radar-derived azimuth and elevation measurements with strict temporal synchronization and is therefore computed entirely prior to flight, the framework could be extended toward online operation if required in future applications. Incremental factor graph solvers, such as iSAM2 [3], provide a natural mechanism for real-time trajectory updates in response to new measurements or disturbances, enabling a future pathway toward limited on-board adaptability while maintaining the measurement-driven reconstruction central to the RES methodology.

Overall, the proposed framework establishes a practical and flexible foundation for UAV-based radar environment simulation, combining precise spatiotemporal synchronization with robust trajectory optimization. Its successful evaluation suggests strong potential for deployment in both research and operational radar systems.

## Figures and Tables

**Table 1:** Angular reconstruction accuracy (RMSE)—scenario 1.

Metric	Value	Units
Azimuth RMSE	0.0165	deg
Elevation RMSE	0.0372	deg

**Table 2:** Angular reconstruction accuracy (RMSE)—scenario 2.

Metric	Value	Units
Azimuth RMSE	0.006	deg
Elevation RMSE	0.059	deg

## Data Availability

The data presented in this study are openly available in the Zenodo repository at https://zenodo.org/records/17377088 (accessed on 29 November 2025), with the identifier DOI: https://doi.org/10.5281/zenodo.17377088.

## Abbreviations

The following abbreviations are used in this manuscript:

Abbreviation	Definition
ADAS	Advanced Driver Assistance Systems
AI	Artificial Intelligence
CA	Constant Acceleration
CV	Constant Velocity
DRFM	Digital Radio Frequency Memory
EKF	Extended Kalman Filter
HIL	Hardware-in-the-Loop
HLA	High Level Architecture
LEO	Low Earth Orbit
ML	Machine Learning
MPC	Model Predictive Control
PRM	Probabilistic Roadmap
QR	QR Decomposition (sparse solver)
RES	Radar Environment Simulator
RRT	Rapidly-Exploring Random Tree
RWR	Radar Warning Receiver
SDR	Software-Defined Radio
TSTP	Time-Synchronized Trajectory Planning
UAV	Unmanned Aerial Vehicle
